# Allergen specific immunotherapy induced multi-organ failure

**DOI:** 10.11604/pamj.2013.14.155.1891

**Published:** 2013-04-23

**Authors:** Aissa Sana, Chaker Ben Salem, Khedher Ahmed, Azouzi Abdelbeki, Sehli Jihed, Ben Saida Imene, Boussarsar Mohamed

**Affiliations:** 1 Intensive Care Unit. Farhat Hached Hospital, Sousse, Tunisia; 2 Department of Clinical Pharmacology. Faculty of Medicine of Sousse, Tunisia

**Keywords:** Allergen specific immunotherapy, multi-organ failure, Allergy, drug reaction, desensitization

## Abstract

Allergen specific immunotherapy (ASI) is a well-documented treatment for allergic asthma, rhinitis and allergy to bee venoms. Immunotherapy with subcutaneous injections of allergens extracts has proved beneficial in reducing symptoms of allergic rhinitis and asthma. Side effects due to specific immunotherapy in short term have been largely documented. These effects were various but were usually mild. Fatal reactions are less frequent. We reported a case of a woman, with a history of allergic asthma under specific desensitization protocol who developed an acute multi-organ failure (MOF) consecutive to administration of ASI (Alustal^®^ Stallergenes SA, France). This fatal reaction has never been described as adverse event of specific immunotherapy. We aimed to describe this dramatic reaction, expose the arguments to define the relationship between the administration of allergen extract and the occurrence of this fatal reaction.

## Introduction

Allergen specific immunotherapy (ASI) is a well-documented treatment for allergic asthma, rhinitis and allergy to bee venoms [1]. Immunotherapy with subcutaneous injections of allergens extracts has proved beneficial in reducing symptoms of allergic rhinitis and asthma [1]. Side effects due to specific immunotherapy in short term have been largely documented. These effects were various (asthma, spasmodic rhinitis, urticaria), but were usually mild [2]. Fatal reactions are less frequent; 1 for 2 to 2.8 million injections [3–5]. We report the case of a woman, with a history of allergic asthma under specific desensitization protocol who developed an acute multi-organ failure (MOF) consecutive to administration of ASI (Alustal^®^ Stallergenes SA, France). This fatal reaction has never been described as adverse event of specific immunotherapy. We aimed to describe this dramatic reaction, expose the arguments to define the relationship between the administration of allergen extract and the occurrence of this fatal reaction.

## Patient and observation

A 17 year-old woman with a history of moderate persistent allergic asthma has benefited from a first desensitization protocol at age 3. The protocol has been interrupted 4 years later because of a mild skin reaction. Her doctor decided to stop the specific immunotherapy. But another doctor restarted a new protocol of desensitization at age 16 against pollens because of a lack of control of his condition. She was never hospitalized, she was receiving no medication and she was doing well one year after she received a new regimen of pneumallergens (Alustal^®^ Stallergenes SA, France). Indeed, 12 hours after initiation of treatment, she complained of abdominal pain, vomiting and diarrhea without fever. Several hours after, she consulted to the emergency department where a surgical emergency was ruled out. She was then admitted to the internal medicine ward. Two days later, she developed an acute respiratory failure and was referred to the intensive care unit where laboratory tests revealed multiorgan failure: liver enzymes, 5000U/L (normal level, 0-37U/L); creatine phosphokinase, 59000U/L (normal level, 10-200U/L); cardiac troponin T, 21ng/ml (normal level, under 0,01ng/ml); leucopenia, 2600/mm^3^ (normal level, 4000-9000/ mm^3^); thrombocytopenia, 13000/ mm^3^ (normal level, 150000-400000/ mm^3^); prothrombin time, 38% (normal level, 70-100%). Chest X-ray demonstrated bilateral interstitial markings with a normal cardiac silhouette. Viral serology was normal for hepatitis A, B, C, D, and E, Epstein-Barr virus and Cytomegalovirus. A skin morbilliform rash and facial edema appeared later. The patient received a fluid resuscitation, platelet and erythrocyte transfusion, steroids and antibiotics. A hypoxic coma occurred on day 4 leading to intubation and mechanical ventilation. Rapidly, she experienced intractable shock and acute renal impairment despite inotropic agents leading to death on day 5.

## Discussion

To our knowledge, this is the first reported case of fata adverse reaction to Allergen Specific Immunotherapy with pneumallergens. Since it was introduced for the first time by Noon and Freeman, the ASI as a treatment for allergic asthma showed its efficiency toward some pneumallergens [2]. However, this treatment, whose mechanisms are not still totally clarified, can sometimes induce adverse reactions [2]. These adverse reactions could be reduced if tight administration protocols, including dosing regimens of administered extracts, rhythm of injections and the way of administration, are adopted.

Despite characterization of susceptibility factors for immunotherapy fatalities, dissemination of earlier survey findings, and publications of immunotherapy practice parameters, the apparent incidence rate of immunotherapy related deaths has not changed in the past 40 years [6].

In 1997, a prospective study was performed in France in order to determine the frequency, the nature, the causes and the consequences of accidents occurring during specific desensitization [2]. 155 accidents out of 151,997 injections made in 19,739 patients during a period of 6 months were registered. The percentage of accidents was low. However, it should be pointed out that the rate was higher for pollen desensitization (114 out of 155 accidents), asthma, spasmodic rhinitis and urticaria were the most frequent manifestations of these systemic reactions (80 per cent of cases) [2]. Only two severe, but not fatal anaphylactic shocks have been reported. In 59 per cent of cases, no obvious explanation could be found for them. The main causes recognized were the administration of excessive doses of extracts, a too long or too short interval between injections, or an improper injection technique [2]. However, after appropriate treatment, desensitization could be continued in 90 percent of cases. Authors stated that specific desensitization, especially with diluted extracts, should not be considered dangerous, but should be carried out with precision and prudence since the errors committed while performing it were responsible for 50 per cent of the accidents recorded [2].

The scarce severe fatal or near-fatal reactions to ASI recorded in literature were related to anaphylactic mechanism [1, 3–5]. In North America, several studies have been conducted over the past 20 years with the purpose of characterizing and estimating the incidence of fatal reactions to immunotherapy [3–5].

The first of these surveys reported 24 fatal reactions that occurred between 1973 and 1984 and estimated that 1 fatal reaction occurred in every 2.8 million injections [3]. Subsequently, Reid et al. described 15 immunotherapy-related fatalities that transpired between 1985 and 1989 and estimated 1 fatality in every 2 million injections. Actually the most recent immunotherapy fatality survey documented 41 fatal reactions between 1990 and 2001, and estimated 1 fatal reaction in every 2.5 million injection [4, 5]. All the fatal reactions reported in these surveys described allergic symptoms (cutaneous signs, urticaria/angioedema, upper airway obstruction, bronchospasm, respiratory failure, hypotension or shock) [1, 3–5].

We report here the first fatal reaction resulting from a multiorgan failure which may probably result from an immunological mechanism. This is probably the consequence of an error of manipulation and/or the escalating of the dosing regimen of the product. The multiorgan failure developed in our patient was imputed to ASI for several reasons. First, according to literature, our patient had some risk factors to develop this adverse reaction. She was on pollen desensitization protocol and she has had a similar reaction to the same drug. Second, this clinical event, including laboratory test abnormalities, with a reasonable time sequence to administration of the drug, is unlikely to be attributed to concurrent disease especially viral or other drugs or chemicals. According to the objective causality assessment by the Naranjo probability scale, ASI-induced acute multi-organ failure was probable in our case [7].

## Conclusion

This observation must alert pulmonologists who widely prescribe this medication. Clinicians must be aware of the potential risk of specific immunotherapy and be interested to observe a very precise protocol of administration of pneumallergens. Patients who have experienced a side effect for specific immunotherapy should be considered at increased risk for future fatal reactions or near-fatal reactions and the physician should consider the risks versus benefits of continuing immunotherapy in this setting.
